# Differential Expression of MicroRNAs in the Colorectal Serrated Neoplasia Pathway and Adenoma–Carcinoma Sequence

**DOI:** 10.1155/grp/1010891

**Published:** 2025-07-14

**Authors:** Takashi Murakami, Hiroyuki Mitomi, Naoki Tsugawa, Yudai Otsuki, Eiji Kamba, Yuichiro Kadomatsu, Takuo Hayashi, Tsuyoshi Saito, Tomoyoshi Shibuya, Takashi Yao, Akihito Nagahara

**Affiliations:** ^1^Department of Gastroenterology, Juntendo University Faculty of Medicine, Tokyo, Japan; ^2^Department of Human Pathology, Juntendo University Graduate School of Medicine, Tokyo, Japan; ^3^Department of Pathophysiological Research and Therapeutics for Gastrointestinal Disease, Juntendo University Faculty of Medicine, Tokyo, Japan

**Keywords:** dysplasia, real-time PCR, sessile serrated adenoma/polyp, submucosal invasion, traditional adenoma

## Abstract

**Background and Aim:** Colorectal carcinogenesis involves two distinct pathways, the serrated neoplasia pathway and adenoma (AD)–carcinoma sequence, whose precursors are sessile serrated lesion (SSL) and traditional AD, respectively. MicroRNAs (miRNAs) regulate gene expression and play a crucial role in colorectal tumorigenesis. This study investigated miRNA expression in the precursors and early invasive carcinomas of the two pathways.

**Methods:** Using real-time reverse transcription polymerase chain reaction, we quantified the expression of miR-20a, miR-21, miR-93, and miR-181b in 127 lesions, including 25 SSLs, 19 SSLs with high-grade dysplasia (SSL-HD), 13 SSLs with submucosal invasive carcinoma (SSL-SC), 19 ADs, 26 ADs with HD (AD-HD), and 25 ADs with SC (AD-SC).

**Results:** In the SSL series, miR-93 (SSL vs. SSL-SC, *p* = 0.038) and miR-181b (SSL vs. SSL-HD/SSL-SC, *p* = 0.013/*p* < 0.001, respectively) levels decreased with tumor progression. In the AD lineage, the expression of miR-20a (AD vs. AD-SC and AD-HD vs. AD-SC, *p* < 0.001), miR-21 (AD vs. AD-HD/AD-SC and AD-HD vs. AD-SC, *p* < 0.001), and miR-181b (AD-HD vs. AD-SC, *p* = 0.020) increased during carcinogenesis. Compared with normal mucosa (baseline), miR-93 expression showed a stepwise increase with tumor progression in the AD lineage, whereas the values did not change during SSL carcinogenesis. In the AD lineage, miR-20a expression increased in early invasive carcinoma but decreased in this phase of the SSL series. Overall, miR-20a, miR-93, and miR-181b levels were significantly lower in SSL-SC than in AD-SC (all *p* < 0.001).

**Conclusions:** These findings indicate that the SSL and AD pathways exhibit distinct miRNA expression dynamics during colorectal tumorigenesis, with the AD lineage showing a progressive increase in oncogenic miRNAs and the SSL series exhibiting selective downregulation or plateauing, particularly in invasive lesions. The differential expression of miR-20a, miR-21, miR-93, and miR-181b was presumed to be related to (epi)genetic alterations among serrated neoplasia and AD–carcinoma routes.

## 1. Introduction

In 2003, Torlakovic et al. [1] described unusual proliferation of colorectal serrated polyps and introduced the terms “sessile serrated polyp” and “sessile serrated adenoma (AD)” to classify these lesions. Currently, the World Health Organization [2] recommends referring to these lesions as sessile serrated lesions (SSLs). SSLs are regarded as early precursors in the serrated neoplasia pathway, leading to colorectal carcinomas characterized by microsatellite instability-high [3]. The serrated pathway is characterized by mutations in *BRAF*, disruptions to the Wnt/*β*-catenin signaling pathway, and widespread methylation of CpG islands [4–7]. This pathway is believed to differ from the traditional AD–carcinoma sequence [8], in which ADs progress to invasive colorectal carcinomas through a series of genetic changes, including mutations in *APC* and *KRAS* [6, 7]. Different apoptotic activities and p21WAF1/CIP1 expressions have been found in SSLs compared with ADs [9]. Moreover, there were differences in the expressions of HIF1*α*, EphB2, and DNA repair proteins and in the frequency of *BRAF*/*KRAS* mutations in those two lesions [10]. Wnt/*β*-catenin signal activation mediated by the methylation of *SFRP4*, *MCC*, and *AXIN2* was dissimilar in the candidates between the serrated pathway and conventional AD routes [11].

MicroRNAs (miRNAs) are small noncoding RNAs consisting of 18–25 nucleotides that play an important role in numerous biological processes, including cell proliferation, differentiation, and apoptosis [12], as well as in the development and progression of colorectal carcinomas [13–22]. The expressions of miR-20a and miR-21 have been evaluated in the tissues of candidates for traditional colorectal carcinogenesis [13–17]. Furthermore, several studies have indicated that miR-181b is implicated in the clinical prognosis of colorectal cancer [18–20]. Alternatively, miR-93, a negative regulator of colorectal carcinogenesis, represses the Wnt/*β*-catenin signaling pathway [21, 22]. In addition, a study on benign colorectal serrated lesions showed a differential expression of miR-21 and miR-181b in SSLs and hyperplastic polyps [23].

To date, very few studies have analyzed the association of specific miRNAs with the serrated neoplasia pathway and traditional AD–carcinoma sequence. This study was aimed at quantitatively assessing the expression of miRNAs, including miR-20a, miR-21, miR-93, and miR-181b, and elucidating the potential roles of the two distinct pathways in colorectal carcinogenesis.

## 2. Methods

### 2.1. Patients and Materials

This study included 127 colorectal polyps (from 125 patients) resected endoscopically or surgically at Juntendo University Hospital and our affiliated hospitals between 2006 and 2014. These included 25 SSLs, 19 SSLs with high-grade dysplasia (SSL-HD), 13 SSLs with submucosal invasive carcinoma (SSL-SC), 19 ADs, 26 ADs with HD (AD-HD), and 25 ADs with SC (AD-SC). Most of the selected lesions were in our previous studies [10, 11].

This study was approved by the Institutional Review Board of Juntendo University School of Medicine (registration #2017166).

### 2.2. Histological Criteria

**Figure 1:**
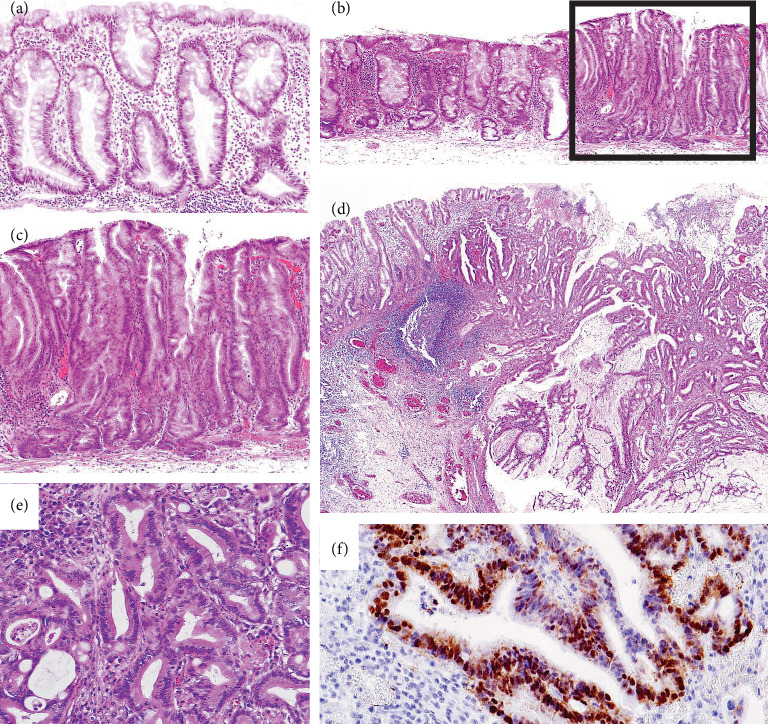
Typical morphology of (a) SSLs, (b, c) SSL-HD, and (d–f) SSL-SC. (a) SSL exhibiting saw-toothed appearance of crypts irregularly dilated at the base (hematoxylin and eosin [HE], original magnification x100). (b) SSL on the left side of the lesion and HD component, indicated by the “square box,” on the right side (HE, original magnification x40). (c) HD component in the SSL shows serrated high-grade dysplasia (HE, original magnification x100). (d) SSL with serrated crypts with partial branching in the lamina mucosae (left side) and carcinoma (right side) with tubulopapillary structure invading the submucosa (HE, original magnification x40). (e) Submucosal invasive carcinoma showing a fused glandular structure (HE, original magnification x100). (f) Carcinoma cells demonstrating nuclear positivity for *β*-catenin immunohistochemistry in the submucosa (immunoperoxidase, original magnification x200).

Histological diagnosis followed the WHO diagnostic criteria for SSLs [2], focusing on features such as crypt serrations, basal crypt dilation, distorted crypts, and growth along the muscularis mucosae. The histologic features of HD were assessed according to a previous description [11] as follows: a tubular, tubulovillous, or fused glandular pattern, mimicking conventional adenomatous HD or a serrated glandular pattern, preserving the serrated or saw-toothed structure with infolding of the crypt epithelium, which consisted of cuboidal and eosinophilic dysplastic cells with substantially larger nuclei and irregular thickening of the nuclear membrane (so-called serrated-type HD). For SSLs with HD or SC, either a normal SSL component at the lesion edge or an abrupt transition to dysplasia or carcinoma within a single tissue fragment was required. Two experienced gastrointestinal pathologists (H.M. and T.Y.) independently reviewed the samples, which were included only if they confirmed the diagnosis. Typical morphologies of the representative cases of the SSL series are shown in Figure 1.

### 2.3. RNA Isolation and Reverse Transcription (RT)

Total RNA was isolated using an RNeasy formalin-fixed paraffin-embedded (FFPE) isolation kit (Qiagen, Hilden, Germany) according to the manufacturer's instructions. The expressions of miR-20a, miR-21, miR-93, and miR-181b were analyzed using a real-time RT-polymerase chain reaction (PCR) scheme for miR quantification according to the protocol of Applied Biosystems (P/N: 4364031). RNA concentration and purity (A260 : A280 > 2.0; A260 : A230 > 1.8) were measured using a NanoDrop 1000 spectrophotometer (Thermo Scientific, Wilmington, Delaware). RT was performed in a 15-*μ*L reaction mixture containing gene-specific stem-loop primers according to the TaqMan MicroRNA Assay protocol (Applied Biosystems, Darmstadt, Germany), 5× RT buffer, 10 mM deoxynucleotide triphosphate (dNTP) (ROTH, Ultrapure dNTP-Set), 200 U/*μ*L RT (Fermentas, RevertAid M-MuLV Reverse Transcriptase, Vilnius, Lithuania), 40 U/*μ*L Ribo-Lock RNase Inhibitor (Fermentas), and 20 ng/*μ*L RNA. The reaction was performed in a peqlab cycler (Primus 25 advanced, MWG Biotech, Ebersberg, Germany) for 30 min at 16°C, 30 min at 42°C, and 5 min at 85°C, and the reaction mixture containing the resulting complementary DNA (cDNA) was stored at 4°C.

### 2.4. Quantitative RT-PCR

The 12-*μ*L real-time PCR mixture contained 5.25 *μ*L cDNA (diluted 1:25), TaqMan Universal PCR master mix (no AmpErase UNG), and 1 *μ*L of each TaqMan MicroRNA assay (hsa-20a/hsa-21/hsa-93/hsa-181b and U6, Applied Biosystems). U6 was used as an endogenous control to normalize the expression levels of the target miRNAs. The relative expression level of each miRNA was calculated using the *Δ*Ct method (Ct miRNA–Ct U6). The primers used in this study are listed in Table 1. The reaction was initiated at 95°C for 10 min, followed by 40 cycles at 95°C for 15 s and 60°C for 1 min. The reactions were carried out using an Applied Biosystems 7500 Fast Real-Time PCR system (Applied Biosystems, Foster City, California).

### 2.5. Statistical Analysis

All statistical analyses were performed using StatView for Windows (Version 5.0, SAS Institute Inc., Cary, North Carolina). Continuous data were compared using the Mann–Whitney *U* test. Categorical analysis of the variables was performed using either the chi-squared test (with Yates correction) or Fisher's exact test, as appropriate. *p* < 0.05 was considered statistically significant.

## 3. Results

### 3.1. Clinicopathological Features

Clinicopathological characteristics of each subtype are summarized in Table 2. The SSL series was predominantly located in the proximal colon (cecum to splenic flexure), whereas the AD lineage was largely located in the distal colon (splenic flexure to rectum) (*p* < 0.001). Grossly, the SSL series was principally sessile, whereas the AD lineage revealed a relatively high proportion of the (semi)pedunculated type (*p* < 0.001). The SSL-HD (mean ± standard deviation [SD], 12 ± 8 mm) was significantly smaller than the AD-HD (17 ± 6 mm; *p* < 0.001).

### 3.2. Expressions of miRNAs

**Figure 2:**
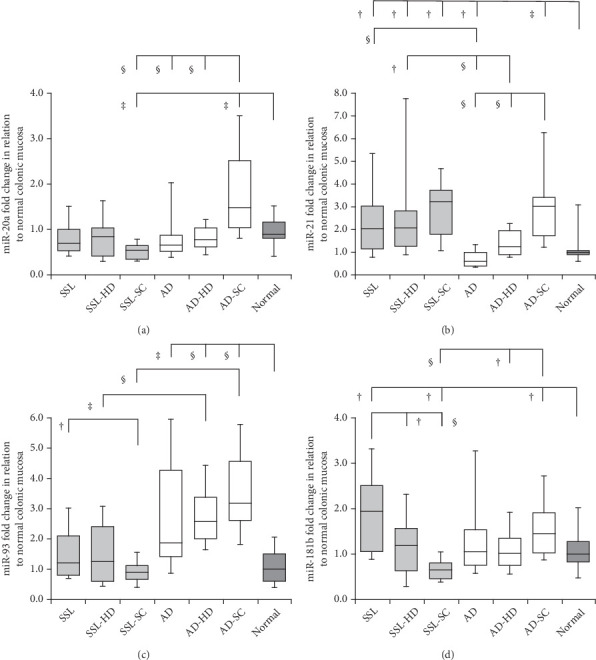
Expression levels of (a) miR-20a, (b) miR-21, (c) miR-93, and (d) miR-181b in the SSL series, AD lineage, and normal mucosa. Box plots show the five-number summary of a set of data, including the minimum score, first (lower) quartile, median, third (upper) quartile, and maximum score. Normal, normal mucosa; ^†^*p* < 0.05; ^‡^*p* < 0.01; ^§^*p* < 0.001.

Detailed data on the expression levels of miR-20a, miR-21, miR-93, and miR-181b in the lesions are presented in Table 3.  Figure 2 illustrates the expression levels of these miRNAs in the SSL series, AD lineage, and normal mucosa.

### 3.3. miR-20a

In the SSL series, the expression levels of miR-20a fluctuated among the subgroups (median ± SD: SSLs: 0.68 ± 3.09, SSL-HD: 0.85 ± 0.76, and SSL-SC: 0.53 ± 0.29). SSL-SC showed significantly lower miR-20a expression than the normal colonic mucosa (0.90 ± 0.43) (*p* = 0.009). A similar trend was observed between SSL-SC and SSLs, with a marginally significant difference (*p* = 0.055). In the AD lineage, the values were significantly higher in AD-SC (1.48 ± 1.46) than in ADs (0.65 ± 1.19; *p* < 0.001), AD-HD (0.77 ± 0.31; *p* < 0.001), and normal mucosa (*p* = 0.004). Comparing the SSL series and AD lineage, miR-20a levels were significantly lower in SSL-SC than in AD-SC (*p* < 0.001).

### 3.4. miR-21

In the SSL series, miR-21 levels were significantly higher in SSLs (2.05 ± 2.04; *p* = 0.016), SSL-HD (2.07 ± 2.68; *p* = 0.019), and SSL-SC (3.22 ± 1.67; *p* = 0.014) than in normal mucosa (0.98 ± 2.72), with no significant differences among the SSL subgroups. In the AD lineage, the expression levels were variable in ADs (0.59 ± 1.24), AD-HD (1.23 ± 0.72), and AD-SC (3.04 ± 2.04). There were significant differences among the groups (ADs vs. AD-HD, ADs vs. AD-SC, and AD-HD vs. AD-SC, *p* < 0.001). The values were significantly lower in ADs (*p* = 0.021) but higher in AD-SC (*p* = 0.001) than in normal mucosa. Comparing the SSL series and AD lineage, the expression levels were significantly higher in SSLs and SSL-HD than in ADs and AD-HD, respectively (SSLs vs. ADs, *p* < 0.001; SSL-HD vs. AD-HD, *p* = 0.026). However, no significant differences were evident between SSL-SC and AD-SC.

### 3.5. miR-93

In the SSL series, miR-93 levels were significantly lower in the SSL-SC (0.92 ± 0.43) than in SSL (1.23 ± 1.22; *p* = 0.038). In the AD lineage, ADs (1.88 ± 2.74; *p* = 0.006), AD-HD (2.59 ± 1.19; *p* < 0.001), and AD-SC (3.18 ± 1.91; *p* < 0.001) showed significantly higher values compared to normal mucosa level (1.00 ± 0.73). The values were significantly lower in SSL-HD and SSL-SC than in AD-HD and AD-SC (SSL-HD vs. AD-HD, *p* = 0.002; SSL-SC vs. AD-SC, *p* < 0.001), with a trend similar to that observed between SSLs and ADs (*p* = 0.051).

### 3.6. miR-181b

In the SSL series, miR-181b expression levels were significantly higher in SSLs (1.96 ± 1.60) than in SSL-HD (1.17 ± 0.89; *p* = 0.013) and SSL-SC (0.66 ± 0.39; *p* < 0.001). Marginal differences were observed between SSL-HD and SSL-SC (*p* = 0.063). Additionally, the values were significantly higher in SSLs (*p* = 0.012) but lower in SSL-SC (*p* = 0.029) than in normal mucosa (0.99 ± 0.91). In the AD lineage, the expression levels were significantly higher in AD-SC (1.45 ± 1.08) than in AD-HD (1.03 ± 0.66; *p* = 0.020) and normal mucosa (*p* = 0.049). The values were significantly lower in SSL-SC than in AD-SC (*p* < 0.001).

## 4. Discussion

This study sheds light on the differential expressions of miR-20a, miR-21, miR-93, and miR-181b in candidates along the serrated and traditional AD–carcinoma routes. In colorectal carcinogenesis, miR-20a regulates *BID*, a gene associated with apoptosis, and affects sensitivity to TRAIL, a TNF superfamily member that induces exogenous apoptosis [14]. Moreover, miR-20a promotes epithelial–mesenchymal transition by suppressing the expression of genes such as *SMAD4* and *GABBR1*, thereby facilitating invasion in colorectal cancer [15]. In this study, high miR-20a expression was observed in AD-SC, suggesting that miR-20a may be involved in the early step of invasion through its association with antiapoptotic mechanisms and epithelial–mesenchymal transition in the AD–carcinoma sequence.

We additionally explored the associations between the expression of miRNAs and methylation of Wnt/*β*-catenin signaling–related genes, *AXIN2* and *MCC*, in the SSL series, including 25 SSLs, 9 SSL-HDs, and 3 SSL-SCs (Table S1). A significant positive correlation was observed between miR-20a expression and *MCC* methylation, suggesting that miR-20a may correlate with Wnt/*β*-catenin signaling activation exclusively in the *MCC*-methylated SSL series. Considerable evidence has shown that miRNAs are implicated in the activation of Wnt/*β*-catenin signaling [13], including overexpression of miR-942 inducing this signaling in AD–carcinoma progression [14]. Further analysis is needed to clarify the relationship between the Wnt/*β*-catenin signaling pathway and miRNA expression in SSL carcinogenesis.

The expression of miR-21 is frequently upregulated in colorectal cancer, and the target genes regulated by miR-21 include *PDCD4*, *RhoB*, and *TGFβR2* [16]. In the present study, miR-21 levels increased from ADs through AD-HD to AD-SC, whereas no significant changes were observed in SSLs, SSL-HD, and SSL-SC. These results suggest that miR-21 is a positive regulator of the AD–carcinoma sequence but not of the serrated pathway. Previous studies have shown that *KRAS* mutations have significant links to the AD series [6, 7]. *KRAS* mutation was infrequent in colorectal cancers with high miR-21 expression compared with those with low-level expression [17]. These findings were discordant with miR-21 expression in the AD series in our study.

miR-93 suppresses CCNB1 protein expression, leading to cell cycle arrest and repression of ERBB2, p21, and VEGF, all of which play important roles in cell proliferation [21]. In colon cancer cells, miR-93 downregulated the Wnt/*β*-catenin pathway, which was confirmed by measuring the expression of *β*-catenin, AXIN, c-Myc, and cyclin D1 in this pathway [22]. Murakami et al. [11] described that upregulation of the Wnt/*β*-catenin signaling pathway, such as nuclear accumulation of *β*-catenin (Figure 1f), contributes to the serrated neoplasia pathway. In the current study, the expression of miR-93 was significantly lower in SSL-SC than in SSLs. Thus, suppression of miR-93 may enhance Wnt/*β*-catenin signaling in carcinogenesis of the serrated pathway.

miR-181a has strong tumor-promoting effects by inhibiting the expression of *WIF-1* and has a potential role in inducing epithelial–mesenchymal transition [18]. miR-181b is associated with colorectal carcinogenesis by targeting *PDCD4* [19], similar to miR-21 [16]. Epigenetic silencing of miR-181b contributes to tumorigenicity in colorectal cancer by targeting *RASSF1A* [20]. In a methylation-specific PCR study using 20 primers, including *APC* and *RASSF1A*, SSLs with higher histological grades showed more extensive methylation than those with lower grades [3]. In our study, miR-181b expression was significantly higher in SSLs but lower in SSL-SC than in normal mucosa, suggesting that the epigenetic silencing mechanism of miR-181b is associated with the early phase of invasion in the serrated pathway. Similar to our results, in a previous study, miR-181b was expressed at higher levels in SSLs than in normal mucosa [23]. Additionally, miR-181b expression levels were significantly higher in AD-SC than in AD-HD and normal mucosa. Accordingly, miR-181b may play diverse roles in the serrated pathway and AD–carcinoma sequence.

**Figure 3:**
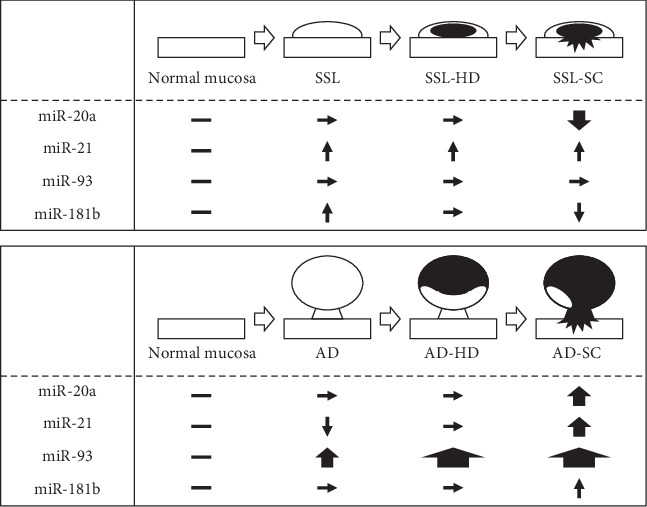
Differences in miRNA expression in the serrated neoplasia pathway and conventional AD–carcinoma sequence. miRNA expression levels are represented as follows: “horizontal line” as baseline values (normal mucosa), “horizontal black arrow” as no significant differences compared with normal mucosa (baseline expression), “thin vertical arrow” as slight differences (*p* < 0.05) compared with normal mucosa, “medium thick vertical arrow” as moderate (*p* < 0.01) differences compared with normal mucosa, and “thick vertical arrow” as marked (*p* < 0.001) difference compared with normal mucosa. Upward vertical arrows indicate an increase in miRNA expression, whereas downward vertical arrows indicate a decrease in miRNA expression. White arrows indicate tumor progression.

We found distinct differences in the expression of miR-20a, miR-21, and miR-181b between the SSL series and AD lineage. A schematic depiction of the differences in the expression of the four miRNAs compared with normal mucosa (baseline expression) between the two routes is shown in Figure 3. The expression of miR-93 showed a stepwise increase during tumor progression in the AD lineage, whereas the values did not change during this step in SSL carcinogenesis. The expression levels of miR-20a increased in the early phase of carcinoma invasion in the AD lineage but decreased in this phase in the SSL series. Recently, miR-21 and miR181a-2 were reported to be associated with carcinogenesis via the serrated pathway [24]. Colorectal cancers with high-level miR21 expression frequently harbor *BRAF* mutations and a CpG island methylator phenotype-high [17], which showed a significant link to the SSL series [5, 7, 10]. This is consistent with the results of our study representing that miR-21 expression is significantly higher in the SSL series than in normal mucosa. Sugai et al. [25] demonstrated no substantial overlap in the expression of miRNAs/messenger RNAs in colorectal cancers with microsatellite instability and precursor SSLs, and they speculated that the network patterns of miRNAs/messenger RNAs change during carcinogenesis in the serrated pathway.

The differential expression levels of miR-20a, miR-21, miR-93, and miR-181b that were observed in this study may have significant clinical implications. These miRNAs may potentially serve as noninvasive biomarkers for the early detection and risk stratification of colorectal neoplasms, particularly those arising from the serrated pathway. Given that serrated lesions—particularly SSLs—are often endoscopically subtle and thus easily missed, the use of circulating or stool-based miRNA assays may further enhance early detection strategies for these malignancies. Furthermore, the expression patterns of these miRNAs may help clinicians differentiate between serrated lesions and conventional ADs, thus facilitating personalized patient surveillance strategies. For example, higher expression levels of miR-21 and miR-181b, which are associated with microsatellite instability-high or high-risk lesions, might flag patients who would benefit from more intensive follow-ups.

Our study focused on a selected panel of four miRNAs (miR-20a, miR-21, miR-93, and miR-181b). Although this targeted approach may not have fully captured the full complexity of the serrated neoplasia pathway, these miRNAs were chosen based on previous evidence suggesting their involvement in colorectal cancer progression—particularly in the contexts of serrated and microsatellite instability [13–23]. Future studies should consider a broader range of miRNAs to further elucidate the molecular basis of colorectal tumorigenesis. For example, miR-31 and miR-135b have both been implicated in the serrated neoplasia pathway—particularly in lesions with high microsatellite instability—high and *BRAF* mutations—whereas miR-143 and miR-145 are commonly associated with AD–carcinoma sequences and *KRAS* or *APC* mutations [26]. Including these miRNAs may enhance our understanding of distinct carcinogenic pathways and support the development of miRNA-based diagnostic or prognostic tools.

This study had several limitations. First, the sample size of each subgroup was relatively small. Additionally, this study focused on the expression of only four miRNAs. Larger cohorts and analyses of more miRNAs are needed to validate the findings and ensure the reproducibility of the differences in miRNA expression. Second, the study relied on FFPE tissue samples, in which RNA was potentially degraded. However, miRNA expression profiles of FFPE tissues closely resemble those of fresh tissues, underscoring the suitability of FFPE tissues as appropriate resources for miRNA analysis [27]. miRNAs are less affected by the fixation/embedding process and are stable (intact) in FFPE samples, and this stability is largely independent of formalin fixation time and duration of tissue block storage [12].

In conclusion, the present study emphasized that the differential expression of miR-20a, miR-21, miR-93, and miR-181b is presumably related to genetic and/or epigenetic variations in candidates between the serrated neoplasia pathway and AD–carcinoma sequence, highlighting their potential roles in carcinogenesis. Our results pave the way for the development of miRNA-based biomarkers for colorectal lesions.

## Figures and Tables

**Table 1:** Primer sequences used for quantitative reverse transcription PCR for miRNAs in this study.

**miRNA target**	**Primer (5**⁣′**→3**⁣′** nucleotides) sequence**
miR-20a	ACUGCAUUAUGAGCACUUAAAG
miR-21	CAACACCAGUCGAUGGGCUGU
miR-93	AAAGUGCUGUUCGUGCAGGUAG
miR-181b	AACAUUCAUUGCUGUCGGUGGG

**Table 2:** Clinicopathological characteristics of colorectal lesions studied.

	**SSLs**	**SSL-HD**	**SSL-SC**	**ADs**	**AD-HD**	**AD-SC**
Lesions (*n*)	25	19	13	19	26	25
Patients (*n*)	25	19	11	19	26	25
Age (years)	63 ± 11 (39–81)	66 ± 10 (54–84)	69 ± 8 (56–84)	68 ± 9 (51–88)	71 ± 10 (44–88)	66 ± 8 (51–79)
Sex						
Male	13 (52%)	12 (63%)	4 (36%)	14 (74%)	14 (54%)	18 (72%)
Female	12 (48%)	7 (37%)	7 (64%)	5 (26%)	12 (46%)	7 (28%)
Location						
Proximal colon	20 (80%)	16 (84%)	13 (100%)	12 (63%)	9 (35%)	2 (8%)
Distal colon	5 (20%)	3 (16%)	0	7 (37%)	17 (65%)	23 (92%)
Macroscopic type						
Sessile	25 (100%)	16 (84%)	11 (85%)	12 (63%)	17 (65%)	14 (56%)
Semipedunculated	0	3 (16%)	2 (15%)	6 (32%)	1 (4%)	3 (12%)
Pedunculated	0	0	0	1 (5%)	8 (31%)	8 (32%)
Size of tumor (mm)	13 ± 6 (3–25)	12 ± 8 (5–36)	13 ± 5 (6–26)	11 ± 6 (4–24)	17 ± 6 (10–32)	17 ± 7 (8–30)

*Note:* Age and tumor size are presented as mean ± SD (range).

**Table 3:** Expression levels of miRNAs in colorectal lesions and normal mucosa.

	**SSLs** **(** **n** = 25**)**	**SSL-HD** **(** **n** = 19**)**	**SSL-SC** **(** **n** = 13**)**	**ADs** **(** **n** = 19**)**	**AD-HD** **(** **n** = 26**)**	**AD-SC** **(** **n** = 25**)**	**Normal mucosa** **(** **n** = 15**)**
miR-20a	0.68 ± 3.09	0.85 ± 0.76	0.53 ± 0.29	0.65 ± 1.19	0.77 ± 0.31	1.48 ± 1.46	0.90 ± 0.43
	(0.24–16.07)	(0.21–3.25)	(0.28–1.35)	(0.26–5.73)	(0.27–1.52)	(0.42–7.42)	(0.16–1.75)
miR-21	2.05 ± 2.04	2.07 ± 2.68	3.22 ± 1.67	0.59 ± 1.24	1.23 ± 0.72	3.04 ± 2.04	0.98 ± 2.72
	(0.53–7.82)	(0.60–9.75)	(0.53–6.64)	(0.21–5.83)	(0.37–3.51)	(0.55–8.17)	(0.54–11.18)
miR-93	1.23 ± 1.22	1.27 ± 1.06	0.92 ± 0.43	1.88 ± 2.74	2.59 ± 1.19	3.18 ± 1.91	1.00 ± 0.73
	(0.57–5.26)	(0.36–3.17)	(0.29–1.71)	(0.48–10.30)	(1.18–6.53)	(0.98–9.81)	(0.31–2.90)
miR-181b	1.96 ± 1.60	1.17 ± 0.89	0.66 ± 0.39	1.07 ± 2.06	1.03 ± 0.66	1.45 ± 1.08	0.99 ± 0.91
	(0.68–8.61)	(0.26–3.84)	(0.35–1.80)	(0.48–8.81)	(0.44–3.12)	(0.54–5.90)	(0.19–3.97)

*Note:* Data are represented as median ± SD (range).

## Data Availability

The data that support the findings of this study are not publicly available because they contain information that could compromise the privacy of research participants but are available from the corresponding author T.M. upon reasonable request.

## Supporting Information

Supporting Information Additional supporting information can be found online in the Supporting Information section. Table S1 Association between *AXIN2* and *MCC* methylation and miRNA expression in serrated lesions.

## References

[1] Torlakovic E., Skovlund E., Snover D. C., Torlakovic G., Nesland J. M. (2003). Morphologic Reappraisal of Serrated Colorectal Polyps. *American Journal of Surgical Pathology*.

[2] Pai R. K., Mäkinen M. J., Rosty C., Nagtegaal I. D., Arends M. J., Odze R. D. (2019). Colorectal Serrated Lesions and Polyps. *WHO Classification of Tumours of the Digestive System*.

[3] Dong S. M., Lee E. J., Jeon E. S., Park C. K., Kim K. M. (2005). Progressive Methylation During the Serrated Neoplasia Pathway of the Colorectum. *Modern Pathology*.

[4] Patil D. T., Shadrach B. L., Rybicki L. A., Leach B. H., Pai R. K. (2012). Proximal Colon Cancers and the Serrated Pathway: A Systematic Analysis of Precursor Histology and BRAF Mutation Status. *Modern Pathology*.

[5] Crockett S. D., Nagtegaal I. D. (2019). Terminology, Molecular Features, Epidemiology, and Management of Serrated Colorectal Neoplasia. *Gastroenterology*.

[6] Jass J. R. (2007). Molecular Heterogeneity of Colorectal Cancer: Implications for Cancer Control. *Surgical Oncology*.

[7] Bosman F., Yan P. (2014). Molecular Pathology of Colorectal Cancer. *Advances in Anatomic Pathology*.

[8] Murakami T., Mitomi H., Yao T. (2018). Distinct Histopathological Characteristics in Colorectal Submucosal Invasive Carcinoma Arising in Sessile Serrated Adenoma/Polyp and Conventional Tubular Adenoma. *Virchows Archiv*.

[9] Mitomi H., Sada M., Kobayashi K. (2003). Different Apoptotic Activity and p21(WAF1/CIP1), but Not p27(Kip1), Expression in Serrated Adenomas as Compared With Traditional Adenomas and Hyperplastic Polyps of the Colorectum. *Journal of Cancer Research and Clinical Oncology*.

[10] Morimoto T., Mitomi H., Saito T. (2014). Distinct Profile of HIF1*α*, PTCH, EphB2, or DNA Repair Protein Expression and BRAF Mutation in Colorectal Serrated Adenoma. *Journal of Gastroenterology and Hepatology*.

[11] Murakami T., Mitomi H., Saito T. (2015). Distinct WNT/*β*-Catenin Signaling Activation in the Serrated Neoplasia Pathway and the Adenoma-Carcinoma Sequence of the Colorectum. *Modern Pathology*.

[12] Pritchard C. C., Cheng H. H., Tewari M. (2012). MicroRNA Profiling: Approaches and Considerations. *Nature Reviews Genetics*.

[13] Zhang N., Hu X., Du Y., Du J. (2021). The Role of miRNAs in Colorectal Cancer Progression and Chemoradiotherapy. *Biomedicine & Pharmacotherapy*.

[14] Huang G., Chen X., Cai Y., Wang X., Xing C. (2017). miR-20a Directed Regulation of BID Is Associated With the TRAIL Sensitivity in Colorectal Cancer. *Oncology Reports*.

[15] Xiao Z., Chen S., Feng S., Li Q., Liu L., Zhang H. (2020). Function and Mechanisms of MicroRNA-20a in Colorectal Cancer. *Experimental and Therapeutic Medicine*.

[16] Ye J. J., Cao J. (2014). MicroRNAs in Colorectal Cancer as Markers and Targets: Recent Advances. *World Journal of Gastroenterology: WJG*.

[17] Mima K., Nishihara R., Yang J. (2016). MicroRNA MIR21 (miR-21) and PTGS2 Expression in Colorectal Cancer and Patient Survival. *Clinical Cancer Research*.

[18] Ji D., Chen Z., Li M. (2014). MicroRNA-181a Promotes Tumor Growth and Liver Metastasis in Colorectal Cancer by Targeting the Tumor Suppressor WIF-1. *Molecular Cancer*.

[19] Liu Y., Uzair-ur-Rehman, Guo Y. (2016). miR-181b Functions as an OncomiR in Colorectal Cancer by Targeting PDCD4. *Protein & Cell*.

[20] Zhao L. D., Zheng W. W., Wang G. X. (2016). Epigenetic Silencing of miR-181b Contributes to Tumorigenicity in Colorectal Cancer by Targeting RASSF1A. *International Journal of Oncology*.

[21] Yang I. P., Tsai H. L., Hou M. F. (2012). MicroRNA-93 Inhibits Tumor Growth and Early Relapse of Human Colorectal Cancer by Affecting Genes Involved in the Cell Cycle. *Carcinogenesis*.

[22] Tang Q., Zou Z., Zou C. (2015). MicroRNA-93 Suppress Colorectal Cancer Development via Wnt/*β*-Catenin Pathway Downregulating. *Tumor Biology*.

[23] Schmitz K. J., Hey S., Schinwald A. (2009). Differential Expression of MicroRNA 181b and MicroRNA 21 in Hyperplastic Polyps and Sessile Serrated Adenomas of the Colon. *Virchows Archiv*.

[24] Peruhova M., Peshevska-Sekulovska M., Krastev B. (2020). What Could MicroRNA Expression Tell Us More About Colorectal Serrated Pathway Carcinogenesis?. *World Journal of Gastroenterology*.

[25] Sugai T., Osakabe M., Niinuma T. (2022). Comprehensive Analyses of MicroRNA and mRNA Expression in Colorectal Serrated Lesions and Colorectal Cancer With a Microsatellite Instability Phenotype. *Genes, Chromosomes and Cancer*.

[26] Eshghifar N., Badrlou E., Pouresmaeili F. (2020). The Roles of miRNAs’ Clinical Efficiencies in the Colorectal Cancer Pathobiology: A Review Article. *Human Antibodies*.

[27] Liu A., Xu X. (2011). MicroRNA Isolation From Formalin-Fixed Paraffin-Embedded Tissues. *Methods in Molecular Biology*.

