# Supplementary material for: Differential Expression of MicroRNAs in the Colorectal Serrated Neoplasia Pathway and Adenoma–Carcinoma Sequence
Source: Gastroenterol Res Pract. 2025 Jul 14;2025:1010891. doi: 10.1155/grp/1010891 (PMC12279426; doi:10.1155/grp/1010891)
Supplement: Supporting Information — Additional supporting information can be found online in the Supporting Information section. Table S1 Association between AXIN2 and MCC methylation and miRNA expression in serrated lesions. [file 1010891.f1.docx]

| **Supplementary Table 1. Association between *AXIN2* and *MCC* methylation and miRNA expression in serrated lesions.** | | | | | | |
| --- | --- | --- | --- | --- | --- | --- |
|  | ***AXIN2* methylation** | | **P value** | ***MCC* methylation** | | **P value** |
|  | **Yes** | **No** |  | **Yes** | **No** |  |
| **miR-20a expression** |  |  |  |  |  |  |
| **High level** | 5 (62%) | 8 (28%) | NS | 9 (56%) | 4 (19%) | 0.036 |
| **Low level** | 3 (38%) | 21 (72%) |  | 7 (44%) | 17 (81%) |  |
| **miR-21 expression** |  |  |  |  |  |  |
| **High level** | 8 (100%) | 23 (79%) | NS | 14 (87%) | 17 (81%) | NS |
| **Low level** | 0 (0%) | 6 (21%) |  | 2 (13%) | 4 (19%) |  |
| **miR-93 expression** |  |  |  |  |  |  |
| **High level** | 6 (75%) | 18 (62%) | NS | 10 (62%) | 14 (67%) | NS |
| **Low level** | 2 (25%) | 11 (38%) |  | 6 (38%) | 7 (33%) |  |
| **miR-181b expression** |  |  |  |  |  |  |
| **High level** | 4 (50%) | 22 (76%) | NS | 9 (56%) | 17 (81%) | NS |
| **Low level** | 4 (50%) | 7 (24%) |  | 7 (44%) | 4 (19%) |  |
| Serrated lesions including 25 SSLs, 9 SSL-HD, and 3 SSL-SC; miRNA expression is defined as high when the values are ≥1-fold compared with normal mucosa, and as low when the values are <1-fold; NS, not significant. | | | | | | |
